# Free Medical Schools

**Published:** 1849-11

**Authors:** 


### ARTICLE II.

*Free Medical Schools.*---The opening lecture for the present session of Rush Medical College, by Prof. N. S. Davis, announces the poliey determined upon by the faculty of the Institution, which is an important reform in the system of Medical Education, contemplating the entire abolition of the professor's ticket fees. To show their sincerity, a commencement of the work was made at once, and to a large class that assembled expecting to pay for all the tickets, as heretofore: those of three of the chairs were given without charge.

The reform is proposed to be carried to other chairs from time to time, as provisions can be made that will justify it, until the design of making the School free is accomplished.

The arguments of Prof. Davis in favor of this policy are quoted below, from the published address, that our readers may judge of ther cogency; with which we close this notice, promising to take up the subject again hereafter:

"The fourth obstacle in the way of a more thorough and complete system of medical education, is the inordinate expense which it imposes on the student. It is a well known fact that few young men possesed of wealth, either in hand or in prospect from their parents, make choice of the responsible and exceedingly toilsome profession of medicine. Hence, four-fifths of all those who commence the study of our profession, are compelled from necessity to practice the most rigid economy, and very many of them pledge a portion of their future earnings, to enable them to comply with the requisitions for graduation.

The evils resulting from this are manifold and serious:

First, It absolutely compels many to resort to those country schols where lecture fees and board are the *cheapest*, let their facilities for imparting practical instruction be ever so limited. And this again fosters another evil, in preventing the schools themselves from depending for patronage wholly on their merits as institutions of sound medical learning.

Second, It compels the student to graduate at the earliest practicable moment—in other words, to make the whole period of his medical studies as short as possible. And in many sections of the country, it compels large numbers to enter into practice before they are even eligible to graduation. Many have supposed that the present high charges for medical instruction tended to limit the number who entered on its study, and thereby to give character and dignity to the profession.—

And this would doubtless be the result to some extent, if the number of medical schools were also limited, and their published terms, in all respects, rigidly adhered to in practice.— But from the very nature of things it is impossible to to build up a professional a istocracy in this country; and hence, so far is the present system from having the effect represented, that it tends directly to cause a multiplication of schools, especially in those locations where they can present to the student the advantages of cheap board, and a slight reduction in the price of tickets, without any reference to other and more important advantages. For it is well known that with the present rate of charges, wherever from fifty to one hundred students can be congregated, it is an object for the Faculty to maintain a school. And yet this very multiplication of schools so increases the competition that very few of them receive as liberal a support as they would with lower charges and less rivalry. Hence we contend that the present system is directly injurious, both in regard to the number and character of the schools themselves.

Third, The expenses at present imposed on the student are entirely out of proportion to the emoluments afterwards resulting from his practice. It is well known that the ordinary period of three years study, and two courses of lectures will seldom require less than an expenditure of from seven hundred to one thousand dollars; while the subsequent actual emoluments from practice, will scarcely equal those resulting from almost any of the mechanic arts. We refer now to the great mass of practitioners and not to the occasional exceptions found in some of our larger towns and cities. This assertion may surprise some of you, but it is the result of careful observation, in a section of country where I am intimately acquainted with a large number of practitioners; some of whom have practiced extensively more than thirty years, and have not accumulated an average of one hundred dollars per annum over their expenses, while many others have really accumulated nothing at all, except an abundance of bad debts.— Hence we may safely assume that it requires an average of from seven to ten years of the practitioner's life to regain the amount he sacrificed, in time and money, to acquire a knowledge of his profession. Again, the legal profession generally receive two or three times as much for the same amount of time and labor, while the expense of gaining admittance into it, is not one quarter as much as into the medical. In whatever light then we view the subject, it appears evident that the cost of a medical education is unreasonably great. Some may contend that we degrade the profession by thus viewing it strictly in the aspect of pecuniary loss and gain. Such tell us that the profession of medicine is, in its very nature, eminently benevolent and humane, and that its members should be actuated in practice by higher and holier motives, than those which look to mere pecuniary rewards. We not only grant all this, but we claim, that whatever the motives of the practitioner may be, the practical result is that a very large proproportion of all his earnings go wholly unpaid—consequently he is practically benevolent far beyond any other class of men, whether he wills it or no. But this, so far from constituting an argument in favor of compelling him to submit to an expensive system of medical instruction, appeals, with overwhelming force, in favor of making his medical education *free* altogether. For that would certainly be a novel mode of reasoning which could show that a body of men ought to pay from seven hundred to one thousand dollars each, for the noble purpose of learning how to spend the rest of their days in acts of benevolence and charity to their fellow men. On the contrary, it would not be difficult to show that the people of every State actually *owe* to the profession far more than enough to educate every student who seeks admission into our ranks. It has been estimated, for instance, that there are forty thousand practitioners in the United States; but grant this to be exaggerated, and that the number actually engaged in practice is only twenty-five thousand; if each of these give only one hundred dollars worth of his labor annually to the poor, gratuitously, (which is very far beneath the actual average) it makes the enormous sum of two million five hundred thousand dollars, which is actually so much indebtedness of the public to the profession. The actual number of medical students attending the various medical schools in our country, is about four thousand; and it will be readily seen that the foregoing indebtedness is doubly sufficient to pay their annual expenses. Hence we contend that every State of this glorious Union justly and honorably owes to the Profession the free education of all its members. But there is still another aspect in which this subject has been viewed.

Dr. Alex. H. Stevens, of New York, late President of the American Medical Association, in an elaborate address before the Medical Society of the State of New York, and the members of the Legislature of that State, entered into an interesting and well founded estimate to show that diminution of the expenses of a medical education, so far as to give every student, properly qualified by preliminary education, free access to our medical colleges, would, by enabling them to devote a longer period of time to study, and consequently to acquire more knowledge and greater skill in the treatment of diseases and injuries, so increase the average duration of life in the community, as to be equivalent to the addition of $1,125,000 to the aggregate wealth of that State.

Hence, he makes a strong appeal to the Legislators of that great State, so to endow her medical colleges that the lecture fees may be entirely abolished. And this is doubtless the only sound and economical policy for every State in the Union to pursue.

Finally, The present expensive system not only contributes much to the general prevalence of all forms of quackery, but by compelling a large proportion of regular students to accept of a very limited amount of medical knowledge—it fosters a class in the profession which serves the same purpose in the medical, that the zoophyte or sponge does in the animal world, viz: to form the connecting link betwen the truly enlightened portion of the profession and the downright quack, thereby rendering it impossible for the community to draw a well defined line of distinction between the two. It contributes to the general prevalence of quackery by inducing many, who, in this free country, are determined to be "doctors," at all hazards, to embrace some one of the numerous special systems that can be learned in a week, or a month, instead of attempting to encounter the embarrassments of a full and regular course. And that it greatly limits the amount of medical knowledge and consequent skill, which, a very large proportion of those who enter the profession, are desirous of obtaining, is too evident to require a word of comment. Hence, in whatever aspect we view the subject, candor compels us to acknowledge that the present system of medical instruction is alike injurious to the schools, unjust and embarrassing to the profession, and greatly detrimental to the best interests of the whole community."

